# Environmental and occupational determinants of myelodysplastic syndrome: A case–control study from Pakistan

**DOI:** 10.1002/cnr2.1580

**Published:** 2021-10-27

**Authors:** Nida Anwar, Aisha Arshad, Naveena Fatima, Sumaira Shaheen, Sumera Bukhari, Tahir Shamsi

**Affiliations:** ^1^ National Institute of Blood Diseases and Bone Marrow Transplantation Karachi Pakistan; ^2^ Cambridge Health Alliance Harvard Medical School Cambridge Massachusetts USA

**Keywords:** environmental exposure, myelodysplastic syndromes, occupational exposure, Pakistan, risk

## Abstract

**Background:**

Myelodysplastic syndromes (MDS) are heterogeneous group of haematopoietic stem cell disorders and have variable reduction in the production of red cells, platelets and mature granulocytes.

**Aim:**

We conducted a case–control study evaluating the environmental and occupational determinants as risk factors of MDS.

**Methods:**

A case–control study was conducted including 150 de novo MDS cases and 450 age and gender‐matched controls. Disease characteristics, sociodemographics and exposure to environmental and occupational determinants were collected through a questionnaire. Chi‐square test was applied to observe association, and binary logistic regression was applied to predict the odds of having MDS.

**Results:**

A total of 600 participants were analysed. Those who were exposed to arsenic (OR 31.81, CI: 19.0–53.0, *P*‐value: .000), benzene (OR 1.564, CI: 1.07–2.27, *P*‐value: .01) using natural source of water (OR 3.563, CI: 2.29–5.53, *P*‐value: .000) and smokers (OR 3.1, *P*‐value: .000) were more likely to have MDS. Unmarried were less likely to acquire MDS than married (OR 0.239, CI: 0.15–0.36, *P*‐value: .000), Sindhi speaking were 1.419 times more likely to have MDS than participants speaking other languages. Uneducated participants were more likely to have MDS than educated and powder milk users were more likely to have MDS than dairy milk users.

**Conclusion:**

Our results revealed that arsenic, use of natural source of water and benzene exposure might lead to higher risk of acquiring MDS. This study would be helpful to understand the aetiology of disease in Pakistani population.

## 1 INTRODUCTION

Myelodysplastic syndromes (MDS) are a heterogeneous group of haematopoietic stem cell disorders characterised by ineffective dysplastic haematopoiesis involving one or more cell lineages, peripheral blood cytopenias and a higher propensity for progression to acute myeloid leukaemia (AML).^1^ MDS is a clonal disorder resulting from complex mutational events in haematopoietic progenitor cells.^1^, ^2^ Patients with this disease have a variable reduction in the production and function of red cells, platelets and mature granulocytes. These quantitative and qualitative abnormalities ultimately result in a variety of systemic consequences such as anaemia, bleeding and increased risk of infection. Conventionally, MDS is classified into different subtypes according to the World Health Organisation (WHO) classifications based on cytopenias, dysplasia, ringed sideroblasts, blast cells, auer rods, along with cytogenetic abnormalities.^3^ The actual incidence of MDS is perhaps higher than reported by cancer databases. Because the nonspecific symptoms of the disease, suspected cases may not undergo early detection and definitive testing, such as bone marrow biopsy, because of co‐morbidities and/or patient preference.^4^

It is well established that like other cancers,^5^, ^6^ the genetic alterations play an important role in MDS as well. Studies have shown that MDS acquires additional genetic mutations that could have impact on the prognosis of disease.^7^ Previous studies have emphasised and demonstrated the possible association of the disease with environmental factors (e.g., chemicals, radiation, tobacco, chemotherapeutic drugs and smoking). The disease has also been associated with certain organic chemicals like pesticides, radiation, petrol, diesel, alcohol, hair dye.^8^ Several risk factors such as high body mass index (BMI) and obesity, less physical activity, autoimmunity are also found to have an association with the occurrence of MDS. However, tea and dietary isoflavone intake are associated with lower risk of acquiring MDS.^9^, ^10^, ^11^, ^12^, ^13^, ^14^, ^15^ Nevertheless, others have identified associations with inherited genetic abnormalities such as Fanconi's anaemia, bloom syndrome, ataxia telangiectasia and other haematological disorders, including paroxysmal nocturnal haemoglobinuria (PNH) and congenital neutropenia.^16^, ^17^ Tobacco smoking is a major source of non‐occupational benzene exposure and it is well documented that cigarette smoke contains large number leukaemia causing agents of which benzene is the most relevant.^18^ Despite several large epidemiological studies and methodologically established in vitro data, there still remains little evidence for a significant contribution of environmental/occupational exposure as a causative risk of MDS. In Pakistan and the surrounding regions, patients do not have access to prompt diagnostic and treatment facilities. Moreover, the characteristics of many patients, such as a low literacy rate, exposure to hazardous chemicals, inadequate access to pasteurised source of milk and refined water, and habitual narcotics use, make them more vulnerable to diseases like MDS at an early age compared with patients in the western world. In this regard, we hypothesised that a roadmap could be drawn to identify risk factors in their daily routines and make them aware of related causal factors that ultimately could lead to life threatening disorders like MDS and thus minimise the disease burden. We therefore, conducted a case–control study evaluating environmental and occupational determinants as risk factors for MDS patients.

## 2 MATERIALS AND METHODS

### 2.1 Study settings and participants

This was a hospital‐based case–control study conducted at National Institute of Blood Diseases and Bone Marrow Transplantation (NIBD & BMT) from January‐2010 to August‐2018.

### 2.2 Cases

*Inclusion criteria*:

- One‐hundred fifty de novo (primary), newly diagnosed, treatment naive MDS cases were enrolled.
- Diagnosis was confirmed according to the WHO 2008 classification.

*Exclusion criteria*:

- Secondary MDS, therapy‐related MDS and patients who had started chemotherapy and other supportive treatment were excluded.

### 2.3 Controls

*Inclusion criteria*:

- Total of 450 age and gender‐matched controls were selected who visited our centre as an attendant of patients. They visited hospital as attendant or patient for other trivial ailments such as diarrhoea.

*Exclusion criteria*:

- Attendants who had personal or family history of blood disorders were excluded.

*Power estimation based on sample size*:

- Power was calculated by G*Power 3.1 software and it was found out 90%.

### 2.4 Data collection tools

The study was approved by the institutional research committee and informed consent was obtained from all participants prior to the collection of epidemiological data. For each case, three controls were enrolled at the hospital while they were visiting for their trivial ailments. On account our centre being one of the referral centres for catering all sorts of blood disorders, we encountered cases from rural and urban areas of Pakistan. All cases and controls were interviewed by using a detailed and well‐structured questionnaire, available in English and translated in local language as well. The questionnaire provided information on (1) sociodemographics variables such as residence, language, educational status, occupation (6 months or more); (2) medical history and habits type of addiction with duration such as (cigarettes, areca nuts, alcohol, and narcotics); (3) environmental exposure such as aspartamine, drinking water source (natural or refined source of water as natural water contains arsenic), dairy source, residential information (whether living near potential hazards such as a nuclear site, chemical waste area, or industrial plant), drug history, X‐ray and radiation exposure, or other radiotherapy exposure for non‐malignant diseases. The habits and environmental exposures history was considered as the exposure to benzene and arsenic.^19^, ^20^, ^21^, ^22^, ^23^, ^24^ Question number 25, 26, 27, 28, 50, 53 in the questionnaire (file attached) were asked from the participants to reveal the exposure to benzene and arsenic. A similar questionnaire was given and analysed parameters for both patient and control to estimate as precisely as possible any exposure to hazardous organic or mineral compounds such as solvents, chemicals, metal, dust particles and potential exposure to viral or bacteriological agents. Further information related to the cytogenetics and haematological profile of the patients was retrieved from their medical records. All questionnaires were reviewed by a group of experts, including clinical research officers.

### 2.5 Statistical analysis

The Statistical Package for the Social Sciences Software (SPSS, version 21) was used for analysis. Qualitative variables, such as marital and education status, residence, language, type of job, milk and water source, smoking status, and arsenic and benzene exposure, cytogenetic profile and subtypes of MDS were presented in terms of frequency and percentages. Chi‐square test was applied to assess the differences in proportions of various determinants between cases and controls. Furthermore, binary logistic regression was applied to estimate odds ratios (OR), 95% confidence interval and *P*‐value to predict the change in the outcome variable when there was a change in independent variable. *P*‐value of ≤.05 was considered as statistically significant. A univariate logistic regression analysis was applied, and OR with 95% confidence intervals (95% CI) were reported to determine the association between exposures and disease occurrence (MDS). First, an association between the different forms of environmental/occupational exposures and MDS was reported in model 0. Afterward, model 1 reported the results after adjusting for sociodemographic variables such as marital status, residence, language, type of job and educational status. Model 2 reported the results after adjusting for the marital status, residence, language, type of job, educational status, water and milk source, smoking and benzene exposure. Finally, model 3 reported the results after adjusting for all covariates plus environmental and occupational exposure to arsenic.

## 3 RESULTS

**FIGURE 1:**
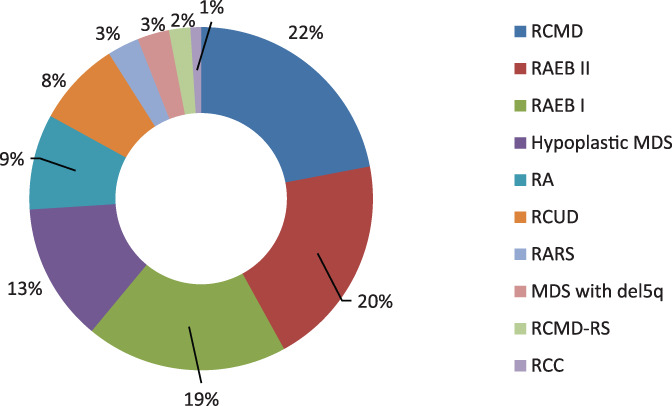
Frequency of MDS patients according to WHO subtype (*N* = 150). RA, refractory anaemia; RAEB I, refractory anaemia with excess blast I; RAEB II, refractory anaemia with excess blasts II; RARS, refractory anaemia with ringed sideroblasts; RCC, refractory cytopenias of childhood; RCMD, refractory cytopenia(s) with multilineage dysplasia; RCMD‐RS, refractory cytopenias with multilineage dysplasia and ringed sideroblasts; RCUD, refractory cytopenias with unilineage dysplasia

**FIGURE 2:**
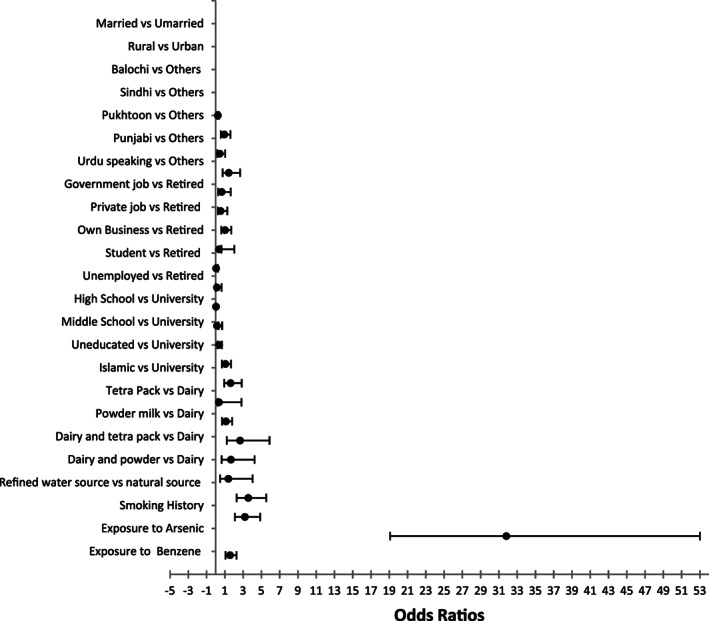
Forest plot showing random effect of each variable against MDS

Six hundred participants were included in the study. Of these, 150 were diagnosed cases of MDS, and 450 were taken as matched controls. Age and gender distribution of cases and controls were statistically identical because of matching (Chi‐squared test *p*‐value = 1.00). The WHO categories of MDS and cytogenetic abnormalities in patients are presented in Figure 1 and Table 1. Majority of the patients (60%) were RCMD (refractory cytopenia(s) with multilineage dysplasia), RAEB II (refractory anaemia with excess blasts II) and RAEB I (refractory anaemia with excess blast I; Figure 1). Cytogenetic analysis was performed in 83 (55%) of patients in which 47 (56.6%) had normal karyotype (Table 1). Chi‐square was applied and significant difference was found between cases and controls regarding marital status, type of job, education status, use of water source, smoking, exposure to arsenic and benzene as shown in Table 2. Binary logistic regression was applied to ascertain the effect of determinants on MDS, and it was found that participants who were exposed to arsenic and benzene, smoking, and those who were using natural source of water were associated with an increased likelihood of exhibiting MDS. Unmarried were less likely to have MDS than married, Sindhi speaking were 1.419 times more likely to have MDS than those who were speaking languages other than Urdu, Punjabi, Balochi and Pukhtoon. Government job officers, private job officers, participants who had their own business and students were less likely to report MDS than retired participants. Uneducated participants were 1.63 times more likely to have MDS than educated. Participants who were using powder milk and combination of tetra pack and dairy milk were more likely to acquire MDS than dairy milk users. The odds ratio, confidence interval and *P*‐value of all the determinants are depicted in Table 3 and Figure 2 represents the odds ratio.

**TABLE 1:** Cytogenetic abnormalities in cases

*N* = 83/150	*n*	%
Normal karyotype	47	56.6
Abnormal karyotype	36	43.4
Complex karyotype	11	13.3
Monosomy 7	7	8.4
Del (5q)	6	7.2
Del (7q)	1	1.2
Trisomy 8	2	2.4
Del (20q)	3	3.6
Trisomy 21	1	1.2
t(1;9) (q11;q34) along with Del(9q)a	1	1.2
Trisomy 8 with Del(7q)	1	1.2
t(6:9) (p23;q23	1	1.2
Monosomy 7,8	1	1.2
Del(9q)	1	1.2

**TABLE 2:** Comparison of characteristics between cases and controls

Characteristics		Group *n* (%) *N* = 600(100)		*P*‐value
	Total	Case 150(25)	Control 450(75)	
Marital status				
Unmarried	297(49.5)	37(24.6)	260(57.8)	<.0001
Married	303(50.5)	113(75.4)	190(42.2)	
Residence				
Urban	513(85.6)	129(86)	384(85.4)	.804
Rural	87(14.4)	21(14)	66(14.6)	
Language				
Urdu	268(44.6)	72(48)	196(43.6)	.037
Balochi	59(9.8)	8(5.3)	51(11.3)	
Sindhi	80(13.3)	27(18)	53(11.8)	
Pukhtoon	37(6.2)	7(4.7)	30(6.7)	
Punjabi	50(8.3)	8(5.3)	42(9.3)	
Others	106(17.6)	28(18.7)	78(17.3)	
Type of job				
Unemployed	216(36)	72(48)	144(32)	<.0001
Government job	27(4.5)	15(10)	12(2.7)	
Private sector job	202(33.7)	27(18)	175(38.9)	
Business owner	69(11.5)	21(14)	48(10.7)	
Student	77(12.8)	8(5.3)	69(15.3)	
Retired	9(1.5)	7(4.7)	2(0.4)	
Educational status				
University	181(30.2)	48(32)	133(29.6)	.001
High school	129(21.5)	15(10)	114(25.3)	
Middle school	195(32.5)	54(36)	141(31.3)	
Uneducated	81(13.5)	30(20)	51(11.3)	
Islamic	14(2.3)	3(2)	11(2.5)	
Milk source				
Dairy	416(69.4)	96(64)	320(71.1)	.113
Tetra pack	119(19.8)	30(20)	89(19.8)	
Powder milk	21(3.5)	7(4.7)	14(3.1)	
Dairy and tetra pack	27(4.5)	12(8)	15(3.3)	
Dairy and powder milk	17(2.8)	5(3.3)	12(2.7)	
Water sources				
Natural sources	358(59.6)	120(80)	238(52.9)	<.0001
Refined sources	242(40.3)	30(20)	212(47.1)	
Smoking history				
Present	343(57)	115(77)	228(51)	.000
Absent	257(43)	35(23)	222(49)	
Arsenic				
Yes	132(22)	103(68.7)	29(6.4)	<.0001
No	468(78)	47(31.3)	421(93.6)	
Benzene				
Yes	239(39.8)	72(48)	167(37.1)	.021
No	361(60.2)	78(52)	283(62.9)	

**TABLE 3:** Binary logistic regression with odds ratio (OR), 95% confidence intervals (CI) and *P*‐Value of MDS determinants in cases and controls

Factors	*B*	Odds ratio	(95%CI)	*P*‐value
Marital status				
Married^a^	–	1	–	.000
Unmarried	−1.430	0.239	(0.158–0.363)	
Residence				
Urban^a^	–	1	–	.841
Rural	−0.54	0.947	(0.557–1.609)	
Language				
Others^a^		1		.062
Urdu	0.023	1.023	(0.615–1.703)	.929
Balochi	0.007	0.437	(0.185–1.034)	.060
Sindhi	0.863	1.419	(0.753–2.674)	.279
Pukhtoon	0.37	0.650	(0.257–1.646)	.363
Punjabi	−0.466	0.531	(0.222–1.267)	.154
Type of Job				
Retired^a^		1		.000
Unemployed	−1.946	0.143	(0.29–0.705)	.017
Government job	−1.030	0.357	(0.62–2.045)	.248
Private sector job	−3.122	0.044	(0.009–0.223)	.000
Business owner	−2.079	0.125	(0.024–0.653)	.014
Student	−3.407	0.033	(0.033–0.006)	.000
Educational status				
University^a^	–	1	–	.001
High school	−1.009	0.365	(0.194–0.686)	.002
Middle school	0.59	1.061	(0.673–1.673)	.798
Uneducated	0.489	1.630	(0.932–2.850)	.087
Islamic	−0.280	0.361	(0.202–2.825)	.677
Milk source				
Dairy^a^	–	1	–	.143
Tetra pack	0.117	1.124	(0.701–1.802)	.629
Powder milk	0.981	2.667	(1.207–5.891)	.015
Dairy and tetra pack	0.511	1.667	(0.654–4.248)	.285
Dairy and powder milk	0.329	1.389	(0.477–4.041)	.547
Water sources				
Refined sources^a^		1		.000
Natural sources	1.271	3.563	(2.293–5.537)	
Smoking history				
Absent^a^		1		
Present	1.163	3.199	(2.100–4.874)	.000
Arsenic				
No^a^	–	1	–	.000
Yes	3.460	31.81	(19.095–53.007)	
Benzene				
No^a^	–	1	–	.019
Yes	0.447	1.564	(1.077–2.272)	

^a^ Reference category.

Univariate logistic regression for model 0 showed that exposure to arsenic was significantly associated with MDS cases (OR = 31.81, 95%CI = 19.095–153.007, *P*‐value <.0001). However, sociodemographics variables (such as marital status, residence, language, type of job, educational status) in model 1 were not significantly associated with having MDS (OR = 0.98, 95%CI = 0.12–0.80, *P* = .03). In model 2, smoking and source of drinking water were significantly associated with having MDS (OR = 1.88, 95%CI = 1.060–3.34, *P* = .03). In model 3, we included all covariates of the study and found that arsenic exposure increased the odds of having MDS (OR = 1.98, 95%CI = 1.09–3.59, *P* = .02; Table 4).

**TABLE 4:** Risk estimation of MDS with exposure to arsenic

Characteristics	Model 0		Model 1		Model 2		Model 3	
Exposure to arsenic Yes No	OR (95% CI)	*P*‐value	OR (95 %CI)	*P*‐value	OR (95 %CI)	*P*‐value	OR (95 %CI)	*P*‐value
	1 31.81 (19.095–53.007)	<.0001	1 0.98 (0.12–0.80)	.03	1 1.88 (1.06–3.34)	.031	1 1.986 (1.098–3.59)	.023

### 3.1 Discussion

A case–control study was conducted to identify a causal relationship between sociodemographic and environmental risk factors and MDS in our cohort of Pakistani patients. In this study, RCMD was the most reported WHO sub‐category of MDS followed by RAEB. Similar findings were reported by another study from Pakistan.^25^ In contrast, refractory anaemia (RA) was most commonly observed subtype in China and Greece.^26^, ^27^ Complex karyotype was most commonly observed in our study (13.3%) contrary to the findings of China^26^ followed by monosomy 7 (8.4%). An Indian study also reported the high frequency of monosomy 7,^28^ contrary to previous studies.^26^, ^29^

Our study revealed that retired participants, low literacy rate, milk source, drinking water source, smoking, exposure to arsenic and benzene were dominant risk factors that were associated with the occurrence of MDS. Our findings were suggestive of the facts that besides genetic factors, socioeconomic profile and exposure to environmental toxins also influence the acquisition of disease.

In a study, arsenic was found to be associated with cytogenetic abnormality^19^, ^20^, ^21^ and benzene was also reported to be a linked to acute leukaemia.^22^, ^23^ In addition the association of benzene, even at low concentrations, with MDS had also been reported in recent years. In one study, Iron et al. found that inflammation in bone marrow was much more prominent in groups exposed to benzene than in non‐exposed groups.^24^, ^30^ One hospital‐based case–control study found a significant association of exposure to benzene and major subtypes of MDS. A number of studies have discussed the association of smoking with MDS^19^, ^31^, ^32^ and in the current study, we had also found the odds of acquisition of MDS among smokers higher than non‐smokers. Arsenic and benzene have been identified in tobacco and it has been reported that smokers have a higher content of arsenic than non‐smokers. Tobacco products may also induce specific chromosomal aberrations, such as deletions in chromosomes five and seven. Other sources of arsenic are air, water, soil, foodstuff and environmental pollutants.^33^, ^34^ Azizullah et al.^35^ and Tariq et al.^36^ reported that a significant proportion of the Pakistani population ingest hazardous substances through drinking water, vegetables, fruits and other edible items and the proportion of chemicals in these substances is higher than the limits proposed by the WHO/Food and Agriculture Organisation (FAO).^34^, ^37^ Similar findings in our study showed that in comparison with the controls, a higher number of MDS cases were using natural sources of water, which could be contaminated with arsenic.^3^ We found one case–control study reporting a significant relationship between different milk sources and aplastic anaemia^37^ but there was no study found that had observed the association of type of milk and water source usage and MDS.

One reported variable in this study that has been linked with the occurrence of MDS is literacy rate. We found that the chance of having MDS decreased with an increasing level of education. Taj et al.^37^ had similar findings in their case–control study of aplastic anaemia patients as our study. It was observed that residents of developing countries that had lower literacy rates and those residing in rural areas had high exposure to pathogenic agents and toxic substances, and also had few medical facilities. This could make them more vulnerable to disease than their educated, urban‐dwelling counterparts.^37^

Retired participants in our study had higher risk of MDS than others and age may be a dominant factor in this case. The mean age of retired participants of cases in our study was 65 ± 6.1 years and it is reported in literature that risk of acquiring MDS increases with age.^38^

Sindhi‐speaking people residing in the rural Sindh areas were more likely to have MDS in our study and it might be due to reason that few lakes situated in Sindh have concentrations of arsenic in water with the range of 35.2–158 μg/L (the mean range is 97.5 μg/L), that is exceeding the permissible limit by WHO.^3^, ^4^, ^39^ Particularly the rural Sindh areas are more affected as they are not aware of the magnitude of problem of using non‐boiled water due to lack of knowledge, beliefs and lower literacy rate as compared to urban areas.

Based on a literature search, a number of studies have reported the serious consequences of alcohol on one's general health. However, alcohol has also been considered as a risk of MDS in many studies but with conflicting results.^40^

## 4 LIMITATION AND STRENGTH

Selection bias is the most commonly reported bias in retrospective case–control studies. However, we have followed strictly the inclusion and exclusion criteria for the enrolment of subjects. One of the limitations of our study was that we have not taken residency‐matched controls. In the present study, we could not develop a hypothesis on the impact of alcohol use on MDS because of the scarcity of data. It was presumed that sociocultural norms would have prevented the subjects in both study groups, that is, cases and controls from honestly revealing their alcohol consumption. In addition, recall bias is always a concern in case–control studies because the data are collected retrospectively. BMI was also not studied because majority of controls were denied to measure height and weight.

Despite the above limitations, this study is important in its unique revelations. To the best of our knowledge, this is one of the few limited studies in region to investigate a wide spectrum of potential risk factors of MDS and the first case–control study conducted in Pakistan. According to our results, arsenic and benzene exposure may increase the risk of acquisition of MDS. However, this sample size is too small to comprehend the aetiology of MDS in Pakistan. Diminutive data are available in this context. In future, further studies are needed to be conducted in this context in different parts of the country which help to identify the risk factors for disease acquisition.

In this case–control study, we found that people in developing countries like Pakistan could be at higher risk than those in developed countries of contracting diseases like MDS due to unrecognised exposure to dangerous environmental and occupational factors. Exposure to arsenic was more prevalent in MDS cases than in controls, and nevertheless Sindhi‐speaking people residing in the rural Sindh areas were more affected having being consuming non‐boiled water as compared to urban areas. A significant association between MDS and low socioeconomic profile was also revealed. This study would add to the existing literature the detrimental impact of environmental and occupational exposure on human health and would also be helpful in understanding the aetiology of MDS in the Pakistani population.

## CONFLICT OF INTEREST

All authors declare no conflicts of interest.

## AUTHOR CONTRIBUTIONS

All authors had full access to the data in the study and take responsibility for the integrity of the data and the accuracy of the data analysis. *Conceptualisation*, N.A., T.S.; *Methodology*, N.A., A.A., N.F., S.S., S.B., T.S.; *Investigation*, N.A., A.A., N.F., S.B., T.S.; *Formal Analysis*, N.A., A.A., N.F., T.S.; *Resources*, T.S.; *Writing—Original Draft*, N.A., A.A., N.F., S.S., S.B., T.S.; *Writing—Review & Editing*, N.A., A.A., N.F., S.S., S.B., T.S.; *Visualization*, N.A., A.A., N.F., S.S., S.B., T.S.; *Supervision*, N.A., A.A., N.F., S.S., T.S.; *Data Curation*, N.A., A.A., N.F., S.S., T.S.; *Project Administration*, N.A., N.F., T.S.; *Validation*, N.A., A.A., N.F., S.S., S.B., T.S.; *Software*, N.F., S.S.

## ETHICAL STATEMENT

The study was approved by National Institute of Blood Diseases and Bone Marrow Transplantation Ethics Committee. The IRB number of this study is NIBD/RD‐161/25‐2017.

## Data Availability

The data that support the findings of this study are available on request from the corresponding author. The data are not publicly available due to privacy or ethical restrictions.
